# Non-invasive Monitor of Effective Chest Compressions with Carotid and Femoral Artery Ultrasound in the Emergency Department

**DOI:** 10.5811/westjem.36710

**Published:** 2025-05-20

**Authors:** Feihong Yang, Hao Zou, Jiaohong Gan, Xia Zhao, Xiaopeng Tu, Cheng Jiang, Jian Xia

**Affiliations:** * Zhongnan Hospital of Wuhan University, Department of Emergency Medicine, Wuhan, 430071, Hubei, People’s Republic of China; † Zhongnan Hospital of Wuhan University, Department of Ultrasound, Wuhan, 430071, Hubei, People’s Republic of China; ‡ Hubei University of Science & Technology, Xianning Medical College, Department of Internal Medicine, Xianning, 437000, Hubei, People’s Republic of China

## Abstract

**Background:**

End-tidal carbon dioxide (EtCO_2_) has been regarded as the gold standard for assessing the effectiveness of cardiopulmonary resuscitation (CPR). However, the clinically observed limitations of EtCO_2_ influenced by ventilation during CPR suggest the need to implement a new, non-invasive hemodynamic monitoring method to evaluate and optimize CPR effectiveness in real time.

**Methods:**

For this prospective study we enrolled 31 cardiac arrest (CA) patients who presented to the emergency department (ED) and 13 healthy volunteers as point-of-care ultrasound (POCUS) controls. Two physicians not involved in the resuscitation team performed POCUS of the bilateral carotid and femoral arteries during chest compression within the first 10 minutes of CPR. The clinical data and presumed CA cause were recorded. We observed the arterial pulse and measured the peak systolic velocity (PSV). The EtCO_2_ values during POCUS were also recorded. We explored the correlation between arterial PSV and EtCO_2_.

**Results:**

The mean age of the patients was 69 ± 2 years, and 22 were male. Of 25 patients who experienced out-of-hospital cardiac arrest, 18 had an average no/low-flow time >30 minutes before ED arrival. Five patients achieved return of spontaneous circulation (ROSC). We found no significant difference in arterial PSV between ROSC and non-ROSC patients. The PSV of the left femoral artery was most consistently and positively correlated with EtCO_2_ in CA patients (R^2^ 0.35, *P*=0.003).

**Conclusion:**

Detection of arterial peak systolic velocity by point-of-care ultrasound, especially of the left femoral artery, might be a feasible method for non-invasive, real-time monitoring of chest compression effectiveness during CPR.

## INTRODUCTION

Cardiac arrest (CA) is a major public health issue with high mortality and disability rates, posing a serious threat to human life. In China, survival rates and good neurological outcomes among CA patients are significantly lower than in developed countries, at only 1.2% and 0.8%, respectively.^1^ Chest compression, the core of cardiopulmonary resuscitation (CPR), is crucial for restoring perfusion to vital organs.^2^ High-quality chest compression can increase blood flow to 25–40% of normal levels,^3^,^4^ promoting myocardial contraction and reducing cerebral ischemic injury. However, there is currently no universally recognized indicator for monitoring the effectiveness and quality of chest compression in Advanced Cardiac Life Support (ACLS).

A rescue team leader or a real-time feedback device can help supervise and optimize the rescuer’s compressions.^5^,^6^ But it is difficult to comprehensively improve the quality and continuity of chest compression when the rescuer lacks systematic and continuous CPR training, or a feedback device may sometimes disrupt rhythmic compressions. Patients’ physiological parameters such as pressure indicators, pulse oxygen saturation (SpO2) waveform, and end-tidal carbon dioxide (EtCO_2_) can also reflect the quality of chest compression.^2^,^4^,^7^ Pressure indicators, including cerebral perfusion pressure and arterial diastolic pressure, are mainly suitable for patients who have already undergone pressure monitoring before CA, for it is difficult to insert intravascular catheters during CPR, and the catheterization process may interrupt the compressions and reduce the number of chest compression fractures (CCF). The SpO2 waveform, the amplitude and peak of which is determined by peripheral arterial blood pressure, is also recommended as an effective monitor.^8^ A study involving 441 CA patients demonstrated that the SpO2 waveform analysis can effectively predict return of spontaneous circulation (ROSC).^9^ However, there are also limitations in monitoring SpO2 waveform in different cases: peripheral hypoperfusion due to long low/no-flow period^10^; venous pulsation due to arteriovenous fistula^11^; abnormal hemoglobin,; skin color changes due to special diseases or poisoning; and others.^12^

High-level EtCO_2_ has been considered a sign of high-quality chest compression and of impending ROSC.^13^,^14^ However, various medical conditions can alter EtCO2 values, even in similar compression conditions, limiting the ability of EtCO2 to accurately evaluate the quality of chest compression. In conditions such as pulmonary embolism or tension pneumothorax that lead to disturbance of the ventilation-perfusion ratio, EtCO_2_ values are always quite low. In contrast, higher EtCO_2_ values could be detected in obstructive airway diseases such as asphyxia, severe asthma, and chronic obstructive pulmonary disease.^14^,^15^

Point-of-care ultrasound (POCUS) is widely available in emergency departments (ED) and has been increasingly used to help manage CA patients during both CPR and the post-resuscitation period.^16^,^17^ Many POCUS protocols have been used to differentiate potential causes, guide further management, and predict prognosis during CPR; these include COACHRED, FEEL, CASA, pulseless electrical activity (PEA) and CAUSE^18^ Ultrasound images of the heart, lung, and aorta can display visible cardiac activity, distinguish between true asystole and fine ventricular fibrillation, and identify the reversible cause of CA. Point-of-care ultrasound can also be used to detect the site and effectiveness of chest compression by directly observing heart motion during CPR.^17^ Some clinical studies on CA patients indicated that Doppler ultrasound of the carotid and femoral arteries, superior to the manual method, could check the pulse faster and more accurately.^19^,^20^

In one prehospital CA case POCUS was recommended for checking the pulse and evaluating the presence of carotid blood flow under chest compression.^21^ An experiment on Landrace pigs found that a wearable Doppler patch to monitor carotid blood flow could provide valuable information about arrhythmia and the quality of CPR, identify ROSC in a timely and effective manner, and assist in managing hemodynamics after ROSC.^22^ In summary, current research indicates that POCUS can be used in CA management to identify causes and monitor ROSC. However, no evidence suggests that POCUS can improve patient prognosis.

**Population Health Research Capsule**

What do we already know about this issue?

*EtCO* *_2_* *is the gold standard for cardiopulmonary resuscitation (CPR) quality assessment but has limitations due to ventilation interference, while ultrasound’s role remains exploratory.*

What was the research question?

*Does real-time arterial POCUS (carotid/femoral peak systolic velocity [PSV]) better predict CPR compression quality than EtCO* *_2_* *?*

What was the major inding of the study?

*Left femoral PSV correlates with EtCO* *_2_* *(R* *^2^* *=0.35, P=0.003) during CPR, suggesting POCUS monitoring feasibility.*

How does this improve population health?

*Arterial POCUS may provide real-time, non-invasive monitoring of CPR effectiveness, potentially improving resuscitation quality and outcomes in CA patients.*

Considering the many limitations in using EtCO2 to monitor chest compression, our goal in this study was to investigate for a correlation between arterial PSV and EtCO2 values during CPR to determine whether real-time arterial POCUS could be used to evaluate the effectiveness of chest compression.

## METHODS

### Design and Setting

This single-center, prospective, observation study was performed in the ED in a Chinese tertiary-care hospital with an annual volume of 120,000 emergency visits. Our rescue area contains seven core beds with monitors and ventilators, as well as one fixed and two portable ultrasound devices, and one transesophageal ultrasound device. All emergency physicians of the resuscitation team have obtained Basic Life Support (BLS) and ACLS certification and mastered rescue skills. The study was approved by the ethics committee of Zhongnan Hospital of Wuhan University (No. LINYANLUN 2022133 dated August 15, 2022) and registered on Clinical Trials (NCT05859516). The study’s principles were in accordance with the Declaration of Helsinki. A waiver of informed consent was granted by the institutional review board.

### Participants

This study, which spanned March 2023–April 2024, included in-hospital cardiac arrest (IHCA) and out-of-hospital cardiac arrest (OHCA) patients who presented to the ED and received BLS/ACLS during the observation period. Due to the availability of manpower, we chose to observe from 8 am to 10 pm every Monday through Friday. The CA patients received mechanical chest compression with the Lund University Cardiopulmonary Assist System (LUCAS) (Lund University, Sweden) and tracheal intubation for ventilation. Patients were excluded if they were <18 years, pregnant, or had been diagnosed with neck, chest or extremities trauma. We also enrolled 13 healthy volunteers as POCUS control.

### Ventilation and EtCO_2_ Record

In accordance with the current guidelines, the LUCAS CPR procedure began with 100–120 chest compressions per minute upon CA patients. Tracheal intubation was simultaneously performed, and an EtCO_2_ monitoring device was connected. The tidal volume was 6 milliliters per kilogram, and the ventilation frequency was 10 breaths per minute. The EtCO_2_ waveform and every minute’s value during the POCUS period were recorded for further analysis.

### Point-of-care Ultrasound Implementation

Two research physicians, proficient in vascular ultrasound after completing one-day Critical Care Ultrasound Study Group (CCUSG) training and one week of intensive practice, were awarded assessment certificates and assigned to performt POCUS in this study. They were solely responsible for performing POCUS and measuring parameters but had no knowledge of the intervention measures or resuscitation outcomes for CA patients. They started to perform POCUS during chest compression within the first 10 minutes of CPR in the ED. The POCUS was performed with Mindray M9 (Shenzhen Mindray Bio-Medical Electronics Co., Ltd, Shenzhen, China). The staff used the L12-4s (12–4 MHz) linear-array probe to obtain vascular long-axis sections with pulse wave Doppler mode in the order of right carotid artery (RCA), left carotid artery (LCA), right femoral artery (RFA), and left femoral artery (LFA).

The POCUS was stopped when the patient reached ROSC or received chest compression for >30 minutes in the ED. The standard for satisfactory POCUS is to clearly display the vessel lumen, the blood-flow time curve, and allow for the measurement of peak systolic velocity (PSV). At least three high-quality, clear images or videos of each vessel were stored and subsequently exported for analysis. The PSV and inner diameter of each vessel were measured three times, and the average values were recorded.

### DATA Collection

The CPR was maintained for at least 30 minutes, until ROSC or the exitus decision was made. The patient’s demographics/health data were collected and recorded on the data collection form, including age, sex, CA location, initial rhythm on presentation, prehospital no/low-flow time, CPR time in the ED, clinical outcome, possible CA cause, and EtCO_2_ values.

### Outcome Measures

The primary outcome of this study was to measure the PSV and vessel diameter in CA patients and healthy volunteers. The secondary outcome was to explore the correlation between arterial PSV and EtCO_2_ values in monitoring the effectiveness and quality of chest compression.

### Statistical Analysis

Descriptive statistics were expressed as medians, standard deviations, interquartile ranges, frequencies, and proportions. For comparisons between groups, we used a paired-samples *t* test, independent samples *t* test in normal data, and rank-sum test in non-normal data. We used simple linear regression to detect the correlation between PSV and EtCO_2_. Statistical calculations were performed at a 95% confidence interval on GraphPad Prism software v 9.5.0.730 (Dotmatics, Ltd, Bishops Stortford, Hertfordshire, England). A *P*-value of <0.05 was considered statistically significant.

## RESULTS

### General Characteristics of Patients

**Figure 1:**
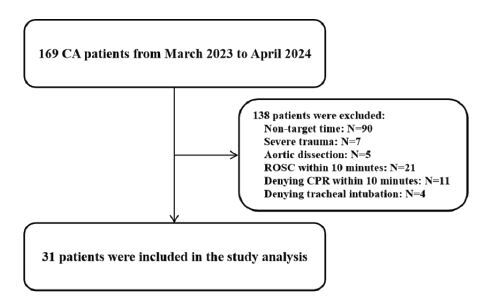
Study population. *CA*, cardiac arrest; *ROSC*, return of spontaneous circulation; *CPR*, cardiopulmonary resuscitation.

This study included 31 CA patients. As shown in Figure 1, we excluded 90 patients who presented at non-target times (night or weekends). An additional 48 patients were excluded for ROSC or denying CPR/tracheal intubation within 10 minutes or who had been diagnosed with severe trauma or aortic dissection. As shown in Table 1, the mean age of the patients was 69±2 years, and 22 (71.97%) patients were males. Twenty-five (80.65%) patients suffered from OHCA, 18 of them with an average no/low-flow time >30 minutes before ED arrival. Most patients had non-shockable initial rhythms (PEA 6.45%, asystole 83.87%) on arrival. Twenty-nine (93.55%) patients were treated with CPR >30 minutes in the ED. Five patients achieved ROSC, and two survived to intensive care unit admission. According to the symptoms, past history, and available laboratory results and imaging examinations, the possible CA cause is shown in Table 1. The EtCO_2_ values during POCUS period were 13.56±1.31 millimeters of mercury (mmHg).

### Comparison of PSV Between CA Patients and Volunteers

**Figure 2:**
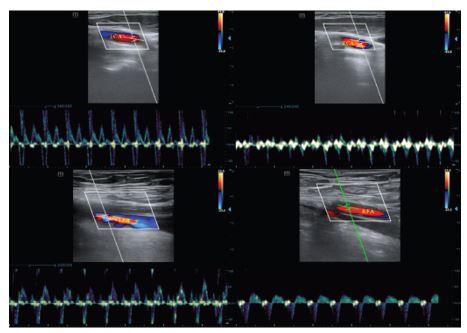
The blood flow of the bilateral carotid and femoral arteries. Top left: LCA; top right: RCA; bottom left: LFA; bottom right: RFA. *LCA*, left carotid artery; *RCA*, right carotid artery; *LFA*, left femoral artery; *RFA*, right femoral artery.

The blood flow of the bilateral carotid and femoral arteries are shown in Figure 2. Peak systolic velocity and inner diameter of the arteries in CA patients and volunteers are compared in Table 2. Among the patients, PSV of the LCA was significantly higher than PSV of the RCA (81.86±8.07 vs 57.12±5.22 centimeters per second [cm/s], *P*=0.02), with similar changes between LCA and LFA (81.86±8.07 vs 59.03±6.26 cm/s, *P*=0.03). Neither was there an obvious difference among the inner diameters of four arteries, which reflected the consistency of vascular resistance. Interestingly, we found that PSV of the RCA in CA patients was lower than in that of volunteers (57.12±5.22 vs 76.00±6.75 cm/s, *P*=0.04), and the inner diameter of the LCA was slightly narrower in CA patients compared with volunteers (0.51±0.03 vs 0.61±0.02 cm/s, *P*=0.03).

### Comparison of PSV Between ROSC and Non-ROSC Patients

We listed PSV and the inner diameter of bilateral carotids of five ROSC patients in Table 3. We also recorded EtCO_2_ values at one minute before ROSC and found that all the values were above average (13.56±1.31 mmHg). There was no correlation between PSV and EtCO_2_ values upon ROSC. We found no significant difference in PSV or inner diameter of any artery between ROSC and non-ROSC patients (Table 4).

### The Correlation Between PSV and EtCO_2_ During CPR

**Figure 3:**
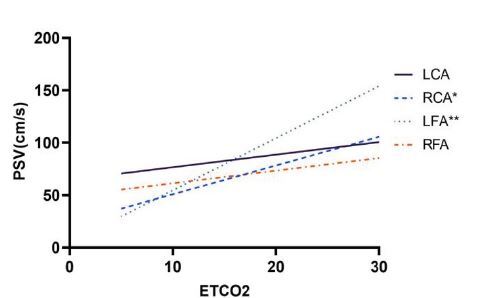
The linear regression lines for peak systolic velocity vs end-tidal carbon dioxide in all cardiac arrest patients. *PSV*, peak systolic velocity; *EtCO_2_*, end-tidal carbon dioxide; *LCA*, left carotid artery; *RCA*, right carotid artery; *LFA*, left femoral artery; *RFA*, right femoral artery.

**Figure 4:**
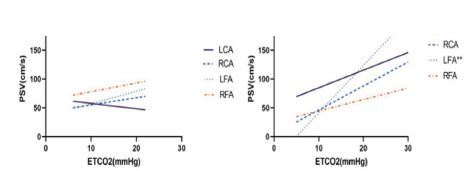
The linear regression lines for peak systolic velocity vs end-tidal carbon dioxide in *OHCA patients. Prehospital CA time < 30 minutes, B. Prehospital CA time ≥ 30 minutes. *OHCA, out-of-hospital cardiar arrest*; PSV*, peak systolic velocity; *EtCO_2_*, end-tidal carbon dioxide; *LCA*, left carotid artery; *RCA*, right carotid artery; *LFA*, left femoral artery; *RFA*, right femoral artery; *CA*, cardiac arrest.

Then we explored the correlation between PSV and EtCO_2_ in CA patients. In Figure 3, the PSV of the LFA was most consistently and positively correlated with EtCO_2_ (R^2^=0.35, *P*=0.003). The PSV of the RCA was also positively correlated with EtCO_2_ (R^2^=0.19, *P*=0.04). Then we divided OHCA patients into two groups based on the prehospital no/low-flow time and found that EtCO_2_ values showed no significant difference between two groups (Table 5). As shown in Figure 4, the PSV of the LFA was most positively correlated with EtCO_2_ (R^2^=0.67, *P*=0.002) in patients with arrest time >30 minutes. No significant correlation between PSV and EtCO_2_ was found in any artery in patients with arrest time <30 minutes.

## DISCUSSION

This was a prospective study to analyze blood flow of the bilateral carotid and femoral arteries during CPR and the first to explore the correlation between arterial PSV and EtCO_2_ on monitoring the effectiveness of chest compression by POCUS in real clinical scenes. Firstly, this study showed that real-time arterial evaluation with POCUS might be a feasible method to monitor the quality of chest compression. Secondly, we found no significant difference in PSV of any artery between ROSC and non-ROSC patients, which suggests the consistency of the quality of chest compression. Thirdly and most interestingly, we found a significantly positive correlation between PSV of LFA and EtCO_2_ in CA patients. Our findings suggest that arterial POCUS, particularly of the LFA, could serve as a novel method for assessing the effectiveness and quality of chest compression. Additionally, we propose a new team composition for ACLS that would include an emergency POCUS physician to enhance resuscitation management.

A growing body of evidence suggests that the quality of CPR, mainly effective and continuous chest compression, is directly related to neurological function recovery and clinical prognosis of CA patients. However, there is still a lack of clinical standards and implementation criteria for quality control of CPR in China. The American Heart Association (AHA) guidelines specify the key points in CPR to ensure high-quality chest compression, including the compression location, posture, depth, frequency, and sufficient rebound.^23^ Chest compression fraction, which is calculated as the ratio of chest compression time to total CPR time, is used to evaluate the continuity of chest compression. The ideal target for CCF in clinical guidelines is 80%.^24^

End-tidal carbon dioxide, considered to reflect cardiac output and coronary perfusion pressure, has been recommended as a non-invasive monitor of CPR effectiveness by AHA guidelines since 2010.^4^,^25^ The EtCO_2_ value, normal 35–45 mmHg, will sharply decrease to <20 mmHg due to mismatched lung ventilation/perfusion in CA patients who recover after ROSC. At present, EtCO_2_ has been mainly used for confirming tracheal intubation, predicting clinical outcome, and identifying ROSC during CPR.^14^,^26^ Research has indicated that different compression depths can lead to changes in EtCO_2_ levels, which are confirmed related to CPR quality.^2^ However, EtCO_2_ can be affected by many factors including the CA cause, pulmonary disease. and therapeutic drugs.^14^,^27^,^28^ Esophageal intubation or complete obstruction of trachea by foreign body may lead to abnormal or no EtCO_2_ waveform.^29^

Asphyxia often has a higher EtCO_2_ value than cardiogenic causes due to CO_2_ retention, while pulmonary embolism shows a lower level.^14^ Patients with previous obstructive pulmonary diseases and receiving intravenous (IV) bicarbonate or vasoactive drugs infusion can show a higher EtCO_2_ level,^27^,^28^ which is also seen in CA patients with acute intracranial hypertension or excessive use of anesthetic drugs. In our study, we found no significant difference of EtCO_2_ values in CA patients with different arrest times, consistent with a generally low EtCO_2_ level in CA patients before ROSC. Differences in the EtCO_2_ values and measured time in various studies have resulted in poor specificity of the EtCO_2_ threshold, leading to uncertain recommended evidence in guidelines.^14^ Some research suggests thresholds of 20 mmHg for detecting ROSC in early CPR,^7^ while others recommend delta EtCO_2_ >20 mmHg with highly specificity for ROSC in PEA patients.^30^ If EtCO_2_ continues to be lower than 10 mmHg after 20 minutes of CPR, it means difficult-to-achieve ROSC.^31^ All these uncertain factors suggest further exploration for the specific EtCO_2_ cut-off point during CPR in comprehensive clinical situations.^4^

In contrast, we found that POCUS centered on arterial blood flow and unaffected by aforementioned factors can effectively monitor the effectiveness and quality of chest compression. Our findings demonstrated successful completion of POCUS examinations on bilateral carotid and femoral arteries during CPR. The PSV of the four arteries showed good consistency of arterial pulsation and blood flow waveforms induced by chest compression. The PSV of the LFA showed the most positive correlation with EtCO_2_ in CA patients. The LFA performs better than carotid arteries in consistency of PSV and the correlation between PSV and EtCO_2_, which may be due to factors such as a smaller impact of chest compression pressure and skin amplitude on the LFA; a relatively flat surface position of the LFA for a larger contact area between the ultrasound probe and skin; and a simpler anatomical position to more easily explore and capture satisfactory images. Thus, we recommend the LFA as the preferred site for POCUS during CPR, considering that it will not affect chest compression, tracheal intubation in the head position, and IV medication in the upper limbs.

Point-of-care ultrasound can assist in evaluating the quality of chest compression, quickly diagnosing the reversible cause of cardiac arrest, monitoring intervention measures and patients’ response, and also predicting the possibility of ROSC and clinical prognosis.^17^,^18^ To evaluate CPR effectiveness, physicians can observe the compression and diastole of the heart directly by POCUS in real time.^17^,^32^ Research has shown that POCUS may lead to an increase in interruption of compressions and CCF decrease.^33^ So, it is recommended that a well-trained physician perform POCUS preferably in the subcostal or apical view within 10 seconds.^34^

Besides confirming reversible causes of non-defibrillation rhythm,^2^,^17^ POCUS can also help rescuers visually establish vascular access and perform pericardiocentesis or thoracentesis to remove the cause of the CA.^34^ A retrospective cohort study showed that POCUS-treated CA patients received more extended CPR and a higher intervention rate compared to those without POCUS.^35^ In recent years, some research has focused on vascular ultrasound during CPR and found that the accuracy of carotid or femoral artery ultrasound in confirming pulse is greatly superior to manual assessment.^19^,^20^ Our research indicates expanding the current use of POCUS in CA patients to monitor and optimize the effectiveness and quality of chest compression through arterial PSV without interrupting compressions and other resuscitation protocols. The latest research has indicated that a small wearable ultrasound device can provide high-quality, real-time, and continuous tissue and vascular ultrasound data. The data is then transmitted to a tablet or smartphone wirelessly for specific evalution.^36^ This provides new insight into the continuing research of POCUS application during CPR.

The PSV reflects the blood velocity passing through the carotid and femoral arteries during systole. Vascular PSV can represent the cardiac ejection to some extent during CPR and, thus, reflect the quality of chest compression. Peak systolic velocity-oriented POCUS, which can be quickly mastered with simple training, is easier to operate than transthoracic echocardiography and transesophageal echocardiography. More importantly, PSV-oriented POCUS does not increase the interruption of chest compression during CPR. There are also certain limitations to the reliability of PSV in reflecting actual arterial flow or perfusion. While the PSV can be quite high, the actual flow can decrease in case of vascular stenosis or obstruction.^37^,^38^ Considering the age and gender of CA populations, atherosclerosis may be common and affect arterial PSV. Therefore, vascular plaque and integrity should be first evaluated when using PSV to reflect the quality of chest compression.

## LIMITATIONS

There are several limitations in our study. First, this was a single-center study with a small sample size carried out only during working hours when the study team was present, which might account for the low R^2^ overall. The small sample size partially resulted in the non-significant correlation between arterial PSV and EtCO_2_ in CA patients with arrest time <30 minutes. Other accounting factors might include the prehospital CA duration, initial heart rhythm at ED admission and reversible etiology, etc. Second, POCUS results are related to the operators’ personal experience. In our study, POCUS was performed by two experienced physicians who had systematically studied the CCUSG course, underwent intensive practice, and passed the assessment in one week, but human confounding factors cannot be completely avoided. Third, the rescue team was not blind to the arterial POCUS while the POCUS physicians had no knowledge of the resuscitation management and outcome. However, this might help improve the quality of chest compression.

Fourth, this study correlated POCUS measurements with the currently controversial parameter, EtCO_2_, which might be problematic in truly reflecting the effectiveness of chest compression. Moreover, in real clinical CPR, it is not feasible to evaluate PSV and other parameters under varying qualities of chest compression. Fifth, due to the limited sample size and results, POCUS was shown to be capable of providing vascular PSV, but we found no impact on resuscitation outcomes. Sixth, in this study two additional physicians were involved in performing POCUS, which might be a challenge in many real-life clinical CPR scenarios. If rescuers take time to perform POCUS, it could lead to increased interruptions in compressions or a lower CCF, both of which are critical for effective CPR. However, our results confirm that arterial POCUS can provide additional data to aid in resuscitation efforts. This finding not only highlights the potential benefits of integrating POCUS into resuscitation protocols but also offers insights for future adjustments in the composition of ACLS team. Therefore, more multicenter studies should be conducted to eliminate various biases and further explore the role of POCUS in improving the effectiveness of CPR.

## CONCLUSION

There are many limitations with regard to the use of EtCO_2_ to monitor CPR quality. Our study supports the feasibility of using peak systolic velocity of the left femoral artery, determined by arterial point-of-care ultrasound, as a non-invasive tool to monitor chest compression effectiveness; however, further research is needed.

## Figures and Tables

**Table 1:** General characteristics of included cardiac arrest patients in the emergency department.

Gender	
Male	22 (71.97%)
Female	9 (29.03%)
**Age**	69±2 years
**Cardiac arrest location**	
OHCA	25(80.65%)
IHCA	6(19.35%)
Prehospital no/low-flow time	
<30 minutes	7(28.00%)
≥30 minutes	18(72.00%)
**Initial emergency department rhythm**	
PEA	2(6.45%)
Ventricular fibrillation/ventricular tachycardia	3(9.68%)
Asystole	26(83.87%)
**CPR time in emergency department**	60[38,80]
<30 minutes	2(6.45%)
≥30 minutes	29(93.55%)
Clinical Outcome	
ROSC	5(16.13%)
Survived to ICU admission	2(6.45%)
Non-ROSC	26(83.87%)
**Possible cause of cardiac arrest**	
Acute myocardial infarction	11(35.48%)
Sepsis	3(9.68%)
Asphyxia	2(6.45%)
Arrhythmia	1(3.23%)
Heart failure	1(3.23%)
Respiratory failure	1(3.23%)
Acute gastrointestinal bleeding	1(3.23%)
Unknown	11(35.48%)
**EtCO** **_2_**	13.56±1.31 mmHg

*OHCA*, out-of-hospital cardiac arrest; *IHCA*, in-hospital cardiac arrest; *PEA*, pulseless electrical activity; *CPR*, cardiopulmonary resuscitation; *ROSC*, return of spontaneous circulation; *ICU*, intensive care unit; *EtCO_2_*, end-tidal carbon dioxide; mmHg, millimeters of mercury.

**Table 2:** Peak systolic velocity and vessel diameter in cardiac arrest patients and volunteers.

	CA patients		Volunteers	
	
	PSV (cm/s)	D (cm)	PSV (cm/s)	D (cm)
LCA	81.86±8.07^a^^b^	0.51±0.03	82.67±7.58	0.61±0.02^d^
RCA	57.12±5.22	0.56±0.03	76.00±6.75^c^	0.62±0.02
LFA	59.03±6.26	0.48±0.03	-	-
RFA	64.27±6.19	0.53(0.46,0.68)	-	-

PSV:

a LCA VS RCA, *P*<0.05,

b LCA VS LFA, *P*<0.05,

c Volunteers vs CA patients, *P*<0.05.

Inner diameter:

d Volunteers vs CA patients, *P*<0.05.

*CA*, cardiac arrest; *PSV*, peak systolic velocity; *D*, inner diameter of vessel; *cm*; centimeters; *LCA*, left carotid artery; *RCA*, right carotid artery; *LFA*, left femoral artery; *RFA*, right femoral artery.

**Table 3:** Peak systolic velocity of bilateral carotids and end-tidal carbon dioxide in five ROSC* patients.

	LCA-PSV (cm/s)	LCA-D (cm)	RCA-PSV (cm/s)	RCA-D (cm)	EtCO_2_ (mmHg)
1	21.39	0.41	59.99	0.56	22
2	140.95	0.70	92.13	0.77	30
3	77.71	0.37	97.14	0.54	15
4	61.67	0.67	54.32	0.48	18
5	24.75	0.50	31.46	0.47	18

*PSV*, peak systolic velocity; *EtCO_2_*, end-tidal carbon dioxide;

* *ROSC*, return of spontaneous circulation; *LCA*, left carotid artery; *D*, inner diameter of vessel; *RCA*, right carotid artery.

**Table 4:** Peak systolic velocity and inner diameter of vessel in ROSC^*^ and non-ROSC patients.

	ROSC (n=5)		non-ROSC (n=26)	
	
	PSV (cm/s)	D (cm)	PSV (cm/s)	D (cm)
LCA	65.29±21.75	0.53±0.07	85.18±8.72	0.51±0.03
RCA	67.01±12.27	0.56±0.05	61.21±8.23	0.55±0.03
LFA	83.27±45.02	0.57±0.03	69.38±8.94	0.46±0.03
RFA	67.57±19.37	0.53±0.06	63.53±6.48	0.56±0.03

*PSV*, peak systolic velocity; *ROSC*, return of spontaneous circulation; *PSV*, peak systolic velocity; *D*, inner diameter of vessel; *LCA*, left carotid artery; *RCA*, right carotid artery; *LFA*, left femoral artery; *RFA*: right femoral artery.

**Table 5:** Peak systolic velocity and end-tidal carbon dioxide in OHCA* patients with different arrest time.

	CA time < 30min (n=7)		CA time ≥ 30min (n=18)	
	
	PSV (cm/s)	EtCO_2_ (mmHg)	PSV (cm/s)	EtCO_2_ (mmHg)
LCA	53.38±12.45	14.71±2.01	101.03±13.82	15.27±2.36
RCA	61.04±11.24		68.33±17.16	
LFA	67.23±10.62		62.67 (22.26,129.1)	
RFA	85.26±13.01		55.08±10.89	

*PSV*, peak systolic velocity; *EtCO_2_*, end-tidal carbon dioxide;

* *OHCA*, out-of-hospital cardiac arrest; *CA*, cardiac arrest; *PSV*, peak systolic velocity; *EtCO_2_*, end-tidal carbon dioxide; *LCA*, left carotid artery; *RCA*, right carotid artery; *LFA*, left femoral artery; *RFA*, right femoral artery.
